# Clinical Spectrum of Complications Induced by Intravesical Immunotherapy of Bacillus Calmette-Guérin for Bladder Cancer

**DOI:** 10.1155/2019/6230409

**Published:** 2019-03-10

**Authors:** Yuqing Liu, Jian Lu, Yi Huang, Lulin Ma

**Affiliations:** Urology Department, Peking University Third Hospital, Beijing, China

## Abstract

Because of its proven efficacy, intravesical Bacillus Calmette-Guérin (BCG) immunotherapy is an important treatment for nonmuscle invasive bladder cancer at high risk of recurrence or progression. However, approximately 8% of patients have to stop BCG instillation as a result of its complications. Complications induced by BCG therapy can have a variety of clinical manifestations. These adverse reactions may occur in conjunction with BCG instillation or may not develop until months or years after BCG cessation. An essential step in the management complications arising from BCG is early establishment of diagnosis, particularly for distant, disseminated, and obscure infections. Therefore we reviewed the literature on the potential complications after intravesical BCG immunotherapy for bladder cancer and provide an overview on the incidence, diagnosis, and treatment modality of genitourinary and systemic BCG-induced complications.

## 1. Introduction

Bladder cancer is the ninth most commonly diagnosed cancer and the thirteenth leading cause of cancer deaths worldwide, with an estimated 429,000 new cases and 165,000 deaths worldwide in 2012 [1]. The majority of patients diagnosed with bladder cancer present with nonmuscle invasive bladder cancer (NMIBC), including carcinoma in situ (CIS) and Ta and T1 stage. Although the gold standard management for NMIBC is complete removal of all papillary lesions via transurethral resection (TUR), there is a high rate of recurrence after the initial surgery. For all high grade and some low grade NMIBC, intravesical immunotherapy of bacillus Calmette-Guérin (BCG) is the one recommended in all the guidelines [2]. The meta-analyses of randomized trials have shown that intravesical maintenance BCG after TUR reduced the recurrence rates significantly [3], and BCG significantly reduces the risk of progression to muscle invasive disease after TUR in patients who receive maintenance BCG [4].

Despite the fact that adjuvant intravesical BCG immunotherapy is significantly more active than surgery alone, a retrospective chart review showed that only 50% of high-risk patients of NMIBC received BCG maintenance therapy as recommended by the EAU and AUA guidelines [5]. The substantial occurrence of complications was regarded as one of the main reasons for poor compliance to BCG therapy. Besides this, it was reported that only 16% of patients on maintenance BCG were able to receive all instillations of the 3-year immunotherapy, mainly because of adverse events [6]. In a more recent report on European Organization for Research and Treatment of Cancer (EORTC) trial 30911, 19% of patients who accepted intravesical BCG had to stop the treatment because of complications, and 29% completed the 3-year immunotherapy [7].

Since the precise immunological mechanism of BCG therapy is still unclear, the pathogeneses of adverse reactions following intravesical BCG instillation have been not fully explained. A controversy exists between the inflammatory hypersensitivity hypothesis, supported by histological findings of granulomas in the absence of microorganisms, and the bacteria invasion hypothesis based on the reports about the positive finding of active bacilli in a variety of tissues. [8] The first essential step following BCG instillation is attachment of live BCG to the urothelium, which brings about recruitment of immune cells to the area, initially granulocytes, followed by macrophages and lymphocytes, and the urinary release of cytokines, which is more pronounced in the case of BCG exposure, reflecting recall immune activation [9]. However, from another point of view, the microbiological identification of* Mycobacterium bovis* (*M. bovis*) from cases of focal disease sites and the fact that ofloxacin administration following intravesical BCG significantly reduced the incidence of BCG-related complications favor argument for direct and hematogenous BCG invasion and infection [10].

Complications induced by BCG can occur and may vary from self-limited irritative voiding symptoms to severe systemic sepsis. According to the results of the largest and most recent published study by the EORTC Genito-Urinary Cancers Group, of the 1316 patients who started BCG, 69.5% reported with local (62.8%) or systemic (30.6%) complications [11]. Chemical cystitis (35.0%) and general malaise (15.5%) were most frequent, and a total of 103 patients (7.8%) stopped treatment because of complications. From a case series and literature review, 282 cases of BCG infection after intravesical instillation were analyzed, and the most common presentations included disseminated (34.4%), genitourinary (23.4%), and osteomuscular (19.9%) infections [8]. The reported incidence of complications induced by intravesical BCG therapy was listed in Table 1.

There are some difficulties in diagnosis of the complications induced by BCG immunotherapy, especially of severe ones. Many complications occur late up to years after cessation of BCG therapy, and symptoms may involve elsewhere beyond the lower urinary tract. Even if there are obvious alarm symptoms induced by BCG, it may be difficult to prove the suspected BCG-related complications. Acid-fast bacilli staining, mycobacterial culture, and polymerase chain reaction (PCR) testing are often negative. Frequently, tissue biopsies are required to evaluate noncaseating granuloma formation, and specimen cultures are helpful to identify the presence of* M. bovis*. The overall rate of positive findings is 48% on microbiological studies (acid-fast bacilli stain, culture, and PCR assay) and 65.5% on tissue biopsy, and identification of granulomatous inflammation on specimen is available in 86.3% of cases [8]. It is a key point that the prognosis of a complication is usually dependent on early treatment initiation, so a high clinical suspicion is important to avoid delay in management. Considering the fact that most complications occur when the patient is already at home, it is quite important that primary care doctors be informed that the patient has received intravesical BCG and be familiar with possible adverse events of BCG, alarming symptoms, and practical knowledge on how to deal with the problems.

In this review, we summarized updated clinical studies and illustrative case reports to provide an overview on the incidence, presentation, diagnosis, and treatments of potential genitourinary and systemic complications resulting from intravesical BCG immunotherapy for NMIBC.

## 2. Genitourinary Complications

### 2.1. Cystitis

Irritative lower urinary tract symptoms are the most common complication after intravesical BCG instillation [12]. Typically, these voiding symptoms can be accompanied by gross hematuria with absence of positive urine cultures. In the EORTC study with BCG instillation, 23.6% cases suffered frequency of more than once per hour, and 22.6% cases complained with macroscopic hematuria [11]. Urine and blood cultures are necessary to exclude bacterial urinary tract infection and/or sepsis.

If the symptoms are considered as the consequence of immune stimulation and the associated inflammatory response, usually accompanied with flu-like illness, spontaneous recovery is expected within 48 hours without intervention [13]. When these symptoms last for a longer time or become increasingly intolerable, symptomatic treatment with spasmolytics, anticholinergics, or nonsteroidal anti-inflammatory drugs is empirically advised. It should be noted that no randomized clinical trials on these drugs even with extended release formulas versus placebo have ever been conducted with the exception of oxybutynin which failed to be proven effective [14].

If bacterial cystitis is diagnosed, antibiotics administration is recommended. Ofloxacin had been proved to diminish BCG-related local complications by a randomized, double-blind clinical trial [15]. Considering the rather high risk of associated bacterial cystitis in persisting voiding symptoms, in a pragmatic way, the antibiotics therapy could start with ofloxacin blindly and then be adjusted when the result of the urine culture is available [16]. Further BCG intravesical therapy should be withheld, not only because BCG microorganisms can be sensitive to antibiotic therapy, but also for avoiding severe inflammatory reactions induced by combination of BCG-induced response and bacterial cystitis. Therefore, BCG instillation should be postponed until completion of a course of culture specific antibiotics. There is little literature to suggest that BCG efficacy is compromised by such adjustments.

### 2.2. Bladder Contracture

Bladder contracture induced by BCG is an uncommon but severe complication [17]. Decreased bladder capacity significantly worsens patients' quality of life and makes patients unable to tolerate BCG instillation. Although published cases of bladder contractures were observed primarily in patients in a maintenance intravesical BCG immunotherapy, there were also cases as early as in the induction phase [18]. Since all patients undergo TUR before BCG therapy, it is difficult to ascertain that BCG alone is the specific cause of bladder contractures [19]. Bladder hydrodistension was reported as an effective treatment option in patients with severely decreased bladder capacity after BCG treatment [20], and the median bladder capacity of five male and one female patients increased from 40 ml (range 30-100 ml) to 200 ml (175-250 ml) within 2 weeks following bladder hydrodistension, with stable volumes during a median follow-up period of 32 months. However, if patients with incapacitating symptoms are refractory to conservative treatment, a short 2-4-week course of oral steroids sandwiched during anti-tuberculous therapy should be considered [21]. It is only the exceptional case that major surgery such as bladder augmentation or cystectomy is required [22].

### 2.3. Bladder Ulcerations

Persisting BCG infections with large inflammatory lesions in bladder can represent tuberculous-like ulcers. In a study of 858 patients with intravesical BCG immunotherapy, thirteen patients developed large bladder ulcerations (10 to 50 mm) [23]. All these patients were male (1.8% of all male patients), all with high grade tumors and seven with invasion to the lamina propria (T1) before BCG treatment. The duration from the initial BCG instillation to the discovery of a visible lesion by routine cystoscopy was 2-34 months (median 8 months). No patient had more than one ulceration or inflammatory lesion despite the fact that seven patients had undergone TURs for multifocal tumors. Urine specimens for mycobacterial culture showed eleven patients were positive, and anti-tuberculosis (anti-TB) therapy (rifampin and isoniazid) given to 8 patients for 6 months resulted in negative urine BCG cultures and resolution of the bladder lesions.

### 2.4. Granulomatous Balanitis

The most widely reported penile complication of BCG is granulomatous balanitis, with symptoms including penile edema, papules, and ulcers, occasionally associated with inguinal lymphadenopathies, typically presenting after multiple cycles of BCG [24]. The time interval between BCG immunotherapy and the onset of skin lesions has been variable, and it ranged from immediately after the instillation to 1 year after the last instillation [25]. The mechanism of infection has been thought to be associated to direct penis inoculation by traumatic urethral catheterization prior to BCG instillation, but the risk factors have been not confirmed. BCG associated granulomatous balanitis lesions were reported previously in 13 cases, including 5 with history of difficult catheterization during BCG instillation. Lymphadenopathy developed in 8 of 9 cases and histological changes (granulomas, necrosis, and ulcer) were reported in 10 cases with adequate information, and 7 of them demonstrated acid-fast bacilli in lymph node biopsies. All cases responded to anti-TB therapy (various combinations of isoniazid, rifampin, and ethambutol without a standard protocol) within 6 to 12 months [26].

### 2.5. Tuberculous Epididymo-Orchitis

Tuberculous epididymo-orchitis, referred to as “BCGitis,” is a rare granulomatous infection resulting from intravesical BCG therapy [27]. It typically manifests as scrotal swelling, pain, dysuria, and fever, commonly occurs within a few weeks of BCG instillation, but may develop as late as 17 years after cessation of BCG therapy [28]. Various distinct gray scale sonographic patterns have been described, including epididymal enlargement or nodules with hypoechoic heterogeneous or homogeneous appearance [29]. Orchitis can demonstrate a miliary sonographic pattern, and multiple intratesticular hypoechoic nodules are thought to be characteristic of tuberculous orchitis [30]. Besides this, other typical findings are also possible, including hydrocele, scrotal skin swelling, intrascrotal calcifications, abscesses, and scrotal sinus formation. Tuberculous epididymo-orchitis can be diagnosed by granulomatous changes, caseous necrosis, and positive acid-fast bacilli in the biopsy specimen. If the development of epididymo-orchitis is delayed from cessation of BCG instillation, a multiplex PCR method can help to confirm whether the BCG strain is the responsible microbe over other mycobacterium species [31]. BCG-induced epididymo-orchitis is usually sensitive to anti-TB therapy, consisting of 300 mg isoniazid and 600 mg rifampin daily for three to six months [29]. To* M. bovis* infection with isoniazid resistance, the regimen includes a fluoroquinolone or an anti-TB aminoglycoside is an option [32]. If the lesion presents persistent pain, swelling, fever, and/or leukocytosis refractory to anti-TB therapy, scrotal exploration and epididymo-orchiectomy may be required.

### 2.6. Prostatitis

Granulomatous prostatitis is one of the most common complications in patients who underwent intravesical BCG immunotherapy, but its incidence is likely to be underestimated by the series of symptomatic cases [33]. In fact, of male patients with BCG instillation, only about 10% developed clinical prostatitis associated with discomfort. More than 40% presented with an elevated serum prostate specific antigen (PSA), and vast majority have histological evidence of BCG granulomatous prostatitis [34].

Despite of the high rate of histological evidence to granulomatous prostatitis, it is difficult to translate this rate to imaging findings due to the limits of radiological technology. In a study of 38 patients evaluated by contrast-enhanced CT scans before TUR and after BCG therapy, abnormal prostatic findings were found in 11 of patients (28.9%), none of whom with any signs or symptoms associated with prostatitis [35]. The MRI characteristics of granulomatous prostatitis after intravesical BCG immunotherapy have not been described extensively and can be represented as a suspicious lesion for harboring prostate cancer [36].

Patients with clinical BCG-induced prostatitis may present with untypical symptoms or signs, including perineal pain, enlarged tender prostate, firm prostate nodules, and elevated prostate specific antigen (PSA). PSA elevation in patients with intravesical BCG is self-limited, and it typically returns to basal levels within 3 months in the absence of maintenance therapy [37]. Prostatic fluid staining for acid-fast bacilli is helpful to identify tuberculous prostatitis. There are sporadic reports of tubercular prostatic abscess as a complication of intravesical BCG immunotherapy [38]. Accompanied with surgical drainage, anti-TB administration is available in controlling the prostatic abscess.

### 2.7. Ureteral Obstruction

Ureteral obstruction is an uncommon complication resulting from intravesical BCG therapy [39]. In most instances, ureteral obstruction is transient and self-limiting after conclusion of BCG therapy. If symptomatic obstruction occurs in the upper urinary tract, a regimen of 300 mg isoniazid and 600 mg rifampin should be given for 3-6 months. Furthermore, temporary drainage is recommended for new onset hydronephrosis. Because of only a few reports on ureteral obstruction following intravesical BCG, there is no evidence for the superiority of either ureteral stenting or percutaneous nephrostomy. In an extremely uncommon case of acute anuria after BCG instillation, the complete obstruction of the vesicoureteral junction resolved spontaneously within 6 days after percutaneous nephrostomy [40].

### 2.8. Kidney Infections

BCG-induced renal infections have involved pyelonephritis and associated renal granulomas. A meta-analysis of 2602 patients revealed a mere 0.1% frequency of renal abscess following BCG instillation [39]. Post-intravesical BCG pyelonephritis may present with systemic symptoms, including persistent low grade fever, chills, fatigue, or weight loss. A hypovascular renal mass may be found by contrast-enhanced CT. Given the fact that post-TUR vesicoureteral reflux has been shown in up to 77% of patients with resection close to the ureteral orifice, the mechanism of renal BCG infections is more likely to be related to reflux of the intravesical BCG rather than hematogenous spread [41]. Renal biopsies can help to establish histopathological diagnosis based on epithelioid cell granulomas; however, acid-fast bacilli may not be present. While granulomas can sometimes be managed conservatively without anti-TB medications, a regimen of 300 mg isoniazid, 600 mg rifampin, and 1200 mg ethambutol daily for 6 months is advised for BCG-induced pyelonephritis or granulomatous masses of the kidney. The treatment modality of genitourinary complications induced by intravesical BCG immunotherapy was listed in Table 2.

## 3. Systemic Complications

Systemic complications occur less frequently (3%-7%) but are usually much more severe. They include disseminated* M. bovis* infection (“BCG-osis”), persistent fever, or any organ involvement beyond genitourinary system. In a review of 282 cases, disseminated infection was defined to consist of sepsis with fever, hypotension, multiorgan failure and coagulopathy, miliary tuberculosis or fever associated with bone marrow and/or liver involvement and/or pulmonary symptoms, accounting for the largest proportion (34.4%) of BCG-induced complications [8]. The mechanism of systemic complication induced by BCG therapy is still under investigation. Although the evidence of viable organisms in tissue supports the theory of systemic spread and dissemination of* Mycobacterium spp.*, it has also been postulated that the systemic response is due to a systemic type IV hypersensitivity reaction to BCG [42]. Diagnosis of disseminated infection is a challenging task due to the diverse array of problems and the lack of evidence from organisms and requires a high index of clinical suspicion on the role of BCG in development of any condition. In fact, the most important clinical clue usually comes from a detailed medical history that elicits prior intravesical BCG therapy.

Clinically, an increased temperature higher than 38.5°C after BCG instillation should arouse a suspicion of disseminated infection, and further intravesical BCG should be withheld. Blood tests often reveal leukopenia and abnormal liver function tests, and chest X-ray may show infiltrates in the lower lung bases [43]. According to EAU guidelines on management for BCG-induced side effects, if the high-grade fever (>38.5°C) persists more than 48 hours, a diagnostic evaluation including urine culture, blood tests, and chest X-ray and a consultation with an infectious disease specialist are necessary, along with prompt treatment with more than two antimicrobial agents. While the evaluation is conducted, no further BCG should be given [44].

### 3.1. Mycotic Aneurysms

The most commonly reported location of mycotic aneurysms after intravesical BCG immunotherapy is aortic (thoracic and abdominal). Moreover, mycotic aneurysms in carotid, iliac, femoral, and popliteal arteries have also been reported. Most reported aneurysms developed within 7-77 months after initial BCG instillation, and more than half of cases presented with rupture of their aneurysms [45]. In a review of 31 aneurysms found in 21 patients after BCG therapy, the most common clinical symptoms were abdominal or back pain (57%), general malaise (52%), fever (38%), and pulsatile or painful mass (19%) [46]. Psoas abscesses have been reported to occur nearly exclusively in patients with mycotic aneurysms and may be caused by the infected aneurysmal leak [47]. Only a few reported cases have shown evidence of typical caseating granuloma in histopathologic examination, so the definitive diagnosis of mycotic aneurysms is based on culture of the aneurysm specimen [45]. Besides this gold standard, microbiological acid-fast bacilli detection and PCR from various tissue samples have been reported [48]. Treatment of mycotic aneurysms includes anti-TB therapy and vascular surgery. The typical surgical repair entails resection of all infected arterial tissues for large aneurysms and revascularization with in situ biological conduits or extra anatomic bypasses. The use of in situ prosthetic conduits was also reported, but the etiology of these aneurysms was not well defined during the surgical repair [46].

### 3.2. Miliary Pulmonary Tuberculosis

Pulmonary complications following intravesical BCG immunotherapy are not common, with an incidence of 0.3%-0.7% of treated patients, presenting as interstitial pneumonitis or miliary dissemination [8, 39]. Symptom onset of miliary dissemination may be abrupt or subacute, and most patients initially present with fever without apparent focus. In a study enrolling 23 male patients after 4 to 16 BCG instillations, 13 patients presented a variety of untypical symptoms, including fever, chills, and weight loss, in addition to respiratory symptoms like cough and dyspnea [49]. Whenever a suspicion of miliary pulmonary tuberculosis is suspected, a chest CT scan should be performed as chest X-ray imaging may miss up to 25% of cases [50]. The detection of acid-fast bacilli positive culture and positive PCR test from sputum or bronchoalveolar lavage can confirm the diagnosis. Considering the fact that 5.4% attributable mortality was observed by a review of the literature [8], it is essential to establish a prompt diagnosis and to initiate anti-TB therapy without delay. Instead of a standardized protocol, a variety of combined isoniazid, ethambutol, streptomycin, or rifampin for 6 to 12 months can provide effective treatment for miliary pulmonary tuberculosis induced by BCG therapy.

### 3.3. Granulomatous Hepatitis

Hepatic complication, typically granulomatous hepatitis, is a rare but fatal complication after intravesical BCG immunotherapy [6, 8]. Granulomatous hepatitis can develop within hours to months or longer following intravesical BCG therapy and present with symptoms and signs of hepatitis, including fever, anorexia, and jaundice [51]. Whereas the diagnosis may be established through detection of acid-fast bacilli in patient's serum or liver tissue by staining techniques, the best differential diagnosis is histopathologic lobular hepatitis by liver biopsy. Initial anti-TB treatment is essential, and the recommended regimen consists of 300 mg isoniazid, 600 mg rifampin, and 1200 mg ethambutol daily for 6 months.

### 3.4. Reactive Arthritis

Osteoarticular complications associated with intravesical BCG therapy are uncommon [52]. Although the mechanisms of rheumatic adverse events of BCG immunotherapy remain not fully clarified, the reactive arthritis is most likely invoked by a systemic immune-mediated response generated by repeated stimuli with live attenuated strains of* M. bovis* and their antigens spreading from the bladder to the circulation [53]. In a systematic review of 89 patients of reactive arthritis following BCG instillation, the symptom of reactive arthritis appeared after a mean number of instillations of 5.8, 13.5 days on average from the last instillation [54]. According to the prevalence, the more frequently affected joints were knee (84.3%), ankle (55.1%), hand (39.3%), wrist (32.6%), and foot (28.1%), and polyarthritis is the most common clinical feature (55.1%) of reactive arthritis related to BCG immunotherapy. Only 20.2% of the patients presented with warning symptoms, including fever, urinary symptoms, unspecific arthralgias, and myalgias. Although the inflammatory joint involvement is induced by an autoimmune reaction, synovial fluid analysis is required to rule out septic arthritis. Therapeutic strategies for BCG reactive arthritis are not well established in the literatures, but the outcome was reported favourable, with no recurrence under nonsteroidal anti-inflammatory drugs, in association with corticosteroids suggested by some authors, and after discontinuation of BCG therapy. For severe cases or those without response to initial therapy, disease-modifying antirheumatic drugs (methotrexate) could be indicated, and addition of isoniazid has also been proposed [55].

### 3.5. Tuberculous Spondylitis

Osteitis is rare secondary to BCG immunization, occurring in less than 37 per 100,000 cases [56]. Updated to 2018, only 23 cases of tuberculous spondylitis or vertebral osteomyelitis following intravesical BCG were reported in the English literature [57]. All these patients were male, with a mean age of 73.2 (35-94) years and with a median interval of 1.3 years (0.5 months-12 years) between BCG instillation and the onset of spondylitis. Chronic back pain was the most common complaint, and serious neurological symptoms might occur as a result of neurosurgical spinal cord decompression [58]. BCG spondylitis is thought to develop by hematogenous spread from the deep pelvic veins to the internal vertebral venous plexuses, which may explain its high incidence in the thoracolumbar spine [59]. The growth of* Mycobacterium* in culture specimens obtained from the infected tissue is the most confirmatory diagnostic test of tuberculous spondylitis; however, histopathological granulomas and acid fast bacilli stain are considered as reference standards for all other diagnostic modalities [60]. Without standard protocol for managing tuberculous spondylitis or vertebral osteomyelitis, most patients were treated by multidrug anti-TB therapy (isoniazid, rifampin, and ethambutol) for an average of 12 months (9-15 months). Surgical intervention will be necessary when the patient has further complications, including spinal cord injury, spinal instability, and abscess formation.

### 3.6. BCG Sepsis

Although a life-threatening septic reaction occurs in an estimated incidence of approximately 1 of 15,000 patients treated with intravesical BCG immunotherapy [39], systemic BCG infection should be suspected in any patient who presents with persistent fever after BCG instillation. Characterized by chills, fever, and hypotension with potential progression to multisystem organ failure, this most dangerous complication is a result of either systemic mycobacterial sepsis and/or a systemic hypersensitivity reaction to BCG [42]. Recommended anti-TB treatment for BCG sepsis consists of 300 mg isoniazid, 600 mg rifampin, and 1200 mg ethambutol daily for 3 to 6 months. In addition, steroid treatment of 40 mg prednisolone should be given intravenously from the beginning of therapy and be tapered gradually after the sepsis is controlled [11]. The treatment modality of systemic complications induced by intravesical BCG immunotherapy was listed in Table 3.

### 3.7. Rare Complications

Central nervous system infections caused by* M. bovis* are very rare; only two cases of cerebellar tuberculomas were reported caused by* M. bovis* BCG in immunocompetent patients without any evidence of systemic dissemination after intravesical BCG immunotherapy [61, 62]. Both cases occurred in elderly male patients with neurological symptoms and signs. Magnetic resonance imaging or contrasted CT scan revealed cerebral nodular foci or mass effect. Although the pathological diagnosis showed chronic granulomatous inflammation from a stereotactic biopsy, the acid-fast smear of the tissue sample was negative. Both patients had a successful response to a 12-month anti-TB medication. In addition, one case used a concomitant steroid therapy, which included dexamethasone transitioned to a 3-month prednisone taper.

There are 15 cases of ocular inflammation after intravesical BCG instillations in the literature. Although cross-reactivity and immunologic reactions are possibly implicated in uveitis after BCG instillations, culture-positive* M. bovis* endophthalmitis was reported in 4 cases which confirmed a direct vitreous invasion after hematogenous dissemination of the microorganism [63]. These cases highlighted a poor ocular outcome despite of an adequate systemic anti-TB therapy, and intravitreal therapy targeted toward M. bovis or additional therapy may be required.

Haemophagocytic syndrome is a very rare but potentially fatal complication of intravesical BCG immunotherapy. This challenging syndrome consists of a sepsis-like state together with variable cytopenia, hepatosplenomegaly, coagulation disorders, and lymphadenopathy; eventually multiple organ failure ensues with a high mortality rate [64]. Early diagnosis is essential to initiate appropriate treatment and improve the survival and quality of life. The immediate aim of therapy is suppression of the increased immune response and specific treatment of the underlying disease [65]. Immunomodulatory regimen depends on the severity of the conditions, including corticosteroids, intravenous immunoglobulins, cyclosporine A, etoposide, antithymocyte globulin, plasma exchange, and splenectomy. Water-soluble steroids such as dexamethasone or methylprednisolone are preferred because of their permeability through blood brain barrier.

## 4. Predisposing Factors Endorsing Complications

### 4.1. Immunosuppression

The incidence of BCG induced adverse events in immunosuppressed patients is unclear because of paucity of data in the literature. In the largest case series of 45 immunosuppressed patients (12 with solid organ transplants, 23 receiving systemic chemotherapy for unrelated cancer, and 10 on steroids for autoimmune disease) treated with intravesical BCG for bladder cancer, BCG therapy was found well-tolerated, and no BCG sepsis was reported [66]. In the literature, only one case of disseminated BCG-induced sepsis was reported 7 years after a 6-week induction course of intravesical BCG therapy for high-grade noninvasive papillary urothelial carcinoma and 1.5 years following immunosuppression for a renal transplantation [67]. Given the fact that this patient's urine and blood mycobacterial culture was positive for the strain of intravesical BCG, the sepsis was most likely caused by organism spreading in the setting of immunosuppression therapy for transplant, rather than systemic hypersensitivity reaction. Therefore, EAU guideline suggests that BCG should be used with caution in immunocompromised patients, despite the comparable incidences of BCG-induced complications reported between patients with and without immunocompromised status [44]. Although BCG is not recommended as vaccination in patients with autoimmune inflammatory rheumatic diseases [68], there is little literature to prove that the safety of intravesical BCG immunotherapy is compromised by administration of synthetic or biological disease-modifying antirheumatic drugs [69].

### 4.2. Geriatric Patients

In the treatment for geriatric patients, age-related deterioration of innate and adaptive immunities and declines in performance status may influence the therapeutic toxicity risk. For elderly patients, the potential side effects and complications of intravesical BCG immunotherapy may be more problematic and not well tolerated. In a study of series of 58 patients, a nearly three-fold higher rate of complications was found in patients older than 70 years compared to younger patients (48.6% vs. 17.6%, respectively) following maintenance BCG therapy [70]. In a recent retrospective study on 200 patients older than 80 years, the elderly patients with multiple comorbidities received a tailored regimen of BCG administration with biweekly scheme, which maintained optimal oncological responses with a significantly lower rate of complications (15% vs. 27%) than that in the elderly patients with better conditions under standard weekly BCG therapy [71].

### 4.3. Chronic Comorbidities

Although chronic comorbidities were regarded as potential predisposing factors of increasing the risk of systemic side effects [42], there was no difference in the incidence of chronic comorbidities such as hypertension, diabetes mellitus, renal insufficiency, and liver disease between 11 patients with BCG infection and 245 others without BCG infection in a review of adjunctive BCG therapy for bladder cancer [8]. In a recent investigation on the influence to intravesical BCG immunotherapy from metabolic syndrome, defined as having three of four components: diabetes mellitus, hyperlipidemia, hypertension, or body mass index ≥ 30kg/m^2^, there was no significant association between metabolic syndrome and the type of BCG failure consisting of recurrence, progression, or intolerance of therapy [72].

## 5. Prevention

Adjunctive BCG administration is imperatively initiated a minimum of 2 weeks after TUR to allow for adequate healing of the urothelium and to prevent systemic absorption of this agent. Moreover, intravesical BCG instillation is contraindicated in patients with visible hematuria, after traumatic catheterization, or with symptomatic urinary tract infection [44]. The potential effect of some antibiotics, notably the fluoroquinolones on BCG viability, and consequent effectiveness should be taken into account.

Reduced dose of intravesical BCG has been reported helpful to decrease the potential toxicity of BCG. In a randomized prospective study, the percentage of no local toxicity in 248 patients with reduced dose of 27 mg intravesical BCG was significantly lower than that in 252 patients with standard dose of 81 mg (33.3% vs. 45.3%) with similar outcomes for recurrence and progression, but the differences in severe systemic toxicity were not significant between reduced dose arm (4.4%) and standard dose arm (3.6%) [73]. However, in a more recent study of 1316 patients, one-third dose was compared to a full dose of BCG given for 1 year or 3 years, and no significant differences in side effects were detected according to dose or duration of BCG treatment in the four arms [11].

Prophylactic medication has been used effectively to decrease the potential toxicity of intravesical BCG. In a randomized prospective, double-blind, multicentre study of 120 patients who received 6 weekly instillations of BCG for urothelial cancer, local side-effects confined to the bladder were significantly lower among those with a 3-day course of isoniazid 300 mg than with placebo (35% vs. 48%) [74]. On the contrary, in a subsequent larger randomized trial, the incidence of cystitis, frequency, hematuria, or severe fever was not reduced significantly in patients treated with BCG and isoniazid, and one case of BCG-induced lung infection developed in this group. Preventive isoniazid therapy also led to treatment discontinuation in significantly more patients (13% vs. 7%) [75]. Beside this, prophylactic 3-day course of prulifloxacin 600 mg was reported to have a significant effect to prevent overall, moderate, and severe adverse events after the fourth intravesical BCG instillation for intermediate- or high-risk NMIBC in a prospective, randomized open-label controlled clinical trial [76]. In addition, a lower proportion that stopped or delayed the full induction course of BCG instillations was noted in patients treated by prior prulifloxacin than in those without prophylactic medication (19% vs. 34%), and the recurrence rates at 3-month or 6-month postoperative cystoscopy were not affected by prulifloxacin treatment. Similarly, in a randomized, prospective, double-blind, placebo controlled, multicenter study, ofloxacin significantly decreased by 18.5% the incidence of moderate and severe adverse events associated with BCG intravesical therapy, particularly class III events, which are primarily associated with patient dropout [15].

## 6. Conclusions

Intravesical BCG immunotherapy has been proved to be effective in the prevention of recurrence and progression of NMIBC for over 40 years; its use is still problematic due to a diverse array of complications that may develop within days to years following the initiation of BCG instillation. Despite these severe complications being uncommon, every physician faced with a patient who has a history of intravesical BCG therapy should be aware of the potential adverse events and the management. Considering the fact that BCG infections and reactions can occur at any organ or position in the body, a detailed medical history provides important clinical value. Because acid-fast staining, culture, and PCR testing are not always positive, tissue biopsies should be performed to evaluate histopathological formation and presence of* M. bovis*. Whereas the most common side effects are self-limited, anti-TB therapy must be used as soon as the diagnosis is established to avert more severe downstream complications. Multidrug anti-TB therapy for 3-12 months may be required along with the rare use of surgery.

## Figures and Tables

**Table 1:** Incidence of complications induced by intravesical BCG immunotherapy for NMIBC [6, 8, 11–13, 17, 23, 26, 27, 34, 39, 52].

Genitourinary complications	Incidence (%)	Systemic complications	Incidence (%)

Cystitis	27-95	Fever (>38.5°C)	2.9
Bladder contracture	< 1	Mycotic Aneurysms	0.7-4.6
Bladder ulceration	1.5	Miliary pulmonary tuberculosis	0.4
Penile lesions*∗*	5.9	Granulomatous hepatitis	0.7-5.7
Tuberculous epididymo-orchitis	0.4	Reactive arthritis	0.5-5.7
Symptomatic prostatitis	10	Tuberculous Spondylitis	3.5
Ureteral obstruction	0.3	BCG sepsis	0.4
Kidney infections	0.3-3.5		

*∗*Penile lesions consisted of nodules, papules, plaques, or ulcers, with or without inguinal lymph node enlargement.

**Table 2:** Treatment modality of genitourinary complications induced by intravesical BCG immunotherapy for NMIBC [13, 15, 17, 20–23, 26, 29, 32, 38, 39].

Genitourinary complications	Initial therapy	Auxiliary treatment	BCG adjustment

Cystitis (irritative voiding symptoms > 48 hours or intolerable)	Spasmolytics, anticholinergics or nonsteroidal anti-inflammatory drugs	Antibiotics administration If bacterial cystitis is diagnosed	Withheld until symptom relieves and antibiotic therapy ends
Bladder contracture	Bladder hydrodistension	Systemic steroids; Exceptionally surgery (bladder augmentation or cystectomy)	Discontinue for decreased bladder capacity
Bladder ulceration	300 mg isoniazid and 600 mg rifampin daily for 6 months	None	Withheld until resolution of the bladder lesion and BCG negative urine
Granulomatous balanitis	Various combinations of isoniazid, ethambutol or rifampin for 6 to 12 months	None	Withheld until the lesion resolves
Tuberculous epididymo-orchitis	300 mg isoniazid and 600 mg rifampin daily for 3 to 6 months	For isoniazid resistance, fluoroquinolones or an anti-TB aminoglycoside; For lesion refractory to anti-TB therapy, scrotal exploration and epididymo-orchiectomy	No further BCG
Symptomatic prostatitis	300 mg isoniazid and 600 mg rifampin daily for 3 to 6 months	Antibiotics (fluoroquinolones) as necessary; Surgical drainage for abscess; Biopsy if no improvement on medication	No further BCG
Ureteral obstruction	300 mg isoniazid and 600 mg rifampin daily for 3 to 6 months	A temporary drainage (ureteral stenting or percutaneous nephrostomy) for hydronephrosis despite conservative therapy	Withheld when onset hydronephrosis; May resume after resolution
Kidney infections	300 mg isoniazid, 600 mg rifampin and 1200 mg ethambutol daily for 6 months	Biopsy if no response to medical treatment	No further BCG

**Table 3:** Treatment modality of systemic complications induced by intravesical BCG immunotherapy for NMIBC [8, 11, 43, 44, 46, 51, 54, 55, 57].

Systemic complications	Initial therapy	Auxiliary treatment	BCG adjustment

Fever (>38.5°C for more than 48 hours)	300 mg isoniazid, 600 mg rifampin, and 1200 mg ethambutol daily for at least 3 months. Plus an empirical non-specific antibiotic to cover Gram-negative bacteria and/or *Enterococcus* with or without steroids.	Treatment adapted to urine culture results.	No further BCG
Mycotic Aneurysms	300 mg isoniazid, 600 mg rifampin and 1200 mg ethambutol daily for 12 months	Surgical resection of aneurysms and revascularization (eg extra anatomic bypass or in situ replacement)	No further BCG
Miliary pulmonary tuberculosis	A variety of combined isoniazid, ethambutol, streptomycin, or rifampin for 6 to 12 months	None	No further BCG
Granulomatous hepatitis	300 mg isoniazid, 600 mg rifampin and 1200 mg ethambutol daily for 6 months	None	No further BCG
Reactive arthritis	Non-steroidal anti-inflammatory drugs ± corticosteroids	Disease-modifying antirheumatic drugs (methotrexate) and/or isoniazid for severe or unimproved cases	BCG can be resumed after benefit-risk assessment till resolution of symptoms; Dose reduction should be considered
Tuberculous Spondylitis	Combined isoniazid, rifampin and ethambutol for 9 to 12 months	Surgical intervention for further complications	No further BCG
BCG sepsis	Emergency admission and intensive care; 300 mg isoniazid, 600 mg rifampin and 1200 mg ethambutol daily for 3 to 6 months; Intravenous 40 mg prednisolone should be given initially and oral steroids taper gradually	Broad-spectrum antibiotics as needed	No further BCG
